# First International External Quality Assessment Study on Molecular and Serological Methods for Yellow Fever Diagnosis

**DOI:** 10.1371/journal.pone.0036291

**Published:** 2012-05-03

**Authors:** Cristina Domingo, Camille Escadafal, Leonid Rumer, Jairo A. Méndez, Paquita García, Amadou A. Sall, Anette Teichmann, Oliver Donoso-Mantke, Matthias Niedrig

**Affiliations:** 1 Centre for Biological Security, Robert Koch Institute, Berlin, Germany; 2 Laboratorio de Virología, Instituto Nacional de Salud, Bogotá, Colombia; 3 Laboratorio de Enfermedades Metaxénicas, Instituto Nacional de Salud, Lima, Perú; 4 Institut Pasteur de Dakar, Dakar, Senegal; Blood Systems Research Institute, United States of America

## Abstract

**Objective:**

We describe an external quality assurance (EQA) study designed to assess the efficiency and accurateness of molecular and serological methods used by expert laboratories performing YF diagnosis.

**Study Design:**

For molecular diagnosis evaluation, a panel was prepared of 14 human plasma samples containing specific RNA of different YFV strains (YFV-17D, YFV South American strain [Brazil], YFV IvoryC1999 strain), and specificity samples containing other flaviviruses and negative controls. For the serological panel, 13 human plasma samples with anti-YFV-specific antibodies against different strains of YFV (YFV-17D strain, YFV IvoryC1999 strain, and YFV Brazilian strain), as well as specificity and negative controls, were included.

**Results:**

Thirty-six laboratories from Europe, the Americas, Middle East, and Africa participated in these EQA activities. Only 16% of the analyses reported met all evaluation criteria with optimal performance. Serial dilutions of YFV-17D showed that in general the methodologies reported provided a suitable sensitivity. Failures were mainly due to the inability to detect wild-type strains or the presence of false positives. Performance in the serological diagnosis varied, mainly depending on the methodology used. Anti-YFV IgM detection was not performed in 16% of the reports using IIF or ELISA techniques, although it is preferable for the diagnosis of YFV acute infections. A good sensitivity profile was achieved in general; however, in the detection of IgM antibodies a lack of sensitivity of anti-YFV antibodies against the vaccine strain 17D was observed, and of the anti-YFV IgG antibodies against a West African strain. Neutralization assays showed a very good performance; however, the unexpected presence of false positives underlined the need of improving the running protocols.

**Conclusion:**

This EQA provides information on each laboratory's efficacy of RT-PCR and serological YFV diagnosis techniques. The results indicate the need for improving serological and molecular diagnosis techniques and provide a follow-up of the diagnostic profiles.

## Introduction

Yellow fever (YF) is a viral disease which is symptomatic in about 5–20% of infected people. In its mildest form YF is characterized by non-specific fever and headache, while the severe form of the disease is characterized by high fever, jaundice, bleeding, and eventually shock and multiple organ failure. Among those who develop severe disease 20–50% may die. To date, there are no antivirals for specific treatment of the infection, and YF vaccination of the population at risk is the best approach to prevent and control the disease [1], [2].

Yellow fever virus (YFV) is transmitted to humans by bites of infected mosquitoes. In the jungle setting, mosquitoes and monkeys maintain the virus *via* an enzootic cycle. The virus can also be transmitted vertically from female mosquitoes to eggs as a maintenance mechanism from one year to the next. A second transmission cycle, the urban cycle, involves humans and the mosquito species *Aedes (Stegomyia) aegypti*. An intermediary cycle is maintained in humid and semi-humid African savannas with viral transmission to humans and to non-human primates through different *Aedes sp.* Mosquito control is not possible in areas of sylvatic transmission, thus eradication of YF is not a plausible option [3].

YF remains an important public health problem for people in endemic regions in Africa and South America and is considered an emerging disease, with a dramatic upsurge in the number of cases in recent years [4]. YF cases occur every year in Africa. For example, during 2011 cases from Senegal, Sierra Leone, Uganda, and Ivory Coast have been reported to the World Health Organization (WHO) (http://www.who.int). In the Americas, the circulation of jungle YF has experienced an unexpected increase since 2008, affecting Argentina, Brazil, Colombia, Paraguay, and Venezuela on the continent and Trinidad and Tobago in the Caribbean. Remarkably, a number cases of urban transmission have been reported in Bolivia [5], and Paraguay has dealt with an outbreak of apparently urban YF [6]. The real incidence of YFV infections worldwide is unknown due to the non-specific nature of the symptoms leading to misdiagnoses, together with insufficient reporting and ground-surveillance, and it is estimated to be over 200,000 cases per year [1].

The clinical diagnosis of YF and the identification of YFV vaccine-associated adverse events (YFVAE) are particularly difficult because of similar symptoms of a wide range of diseases (e.g. dengue, leptospirosis, viral hepatitis, malaria, hemorrhagic viral diseases), therefore laboratory confirmation is essential. As criteria for laboratory YF diagnosis, WHO recommends the detection of YFV-specific IgM or a fourfold or greater rise in serum IgG levels in the absence of recent YF vaccination and a negative diagnosis for other flaviviruses. Isolation of YFV, positive post-mortem liver histopathology, detection of YFV antigen in tissues by immunohistochemistry, or detection of YFV genome in blood or organs by RT-PCR also confirm the presence of YFV infection [7]. Recently, we have reported the detection of YFV-17D genome in the urine of healthy YF vaccinees and vaccinees with serious post- vaccination adverse events, which seems very promising for the investigation in outbreak situations by non-invasive sampling methods [8].

Molecular methods for the detection of the viral genome offer a rapid, sensitive, and highly specific alternative for early serological diagnosis during the viraemic phase of infection or in post-mortem tissues. After the viraemic phase the use of serological methods represents a good option to confirm the infection, but generally two samples are required to be taken at least two weeks apart. Serological diagnosis commonly includes the use of haemagglutination, ELISA, indirect immunofluorescence (IIF) and seroneutralization assays, but flavivirus serological cross-reactions constitute a major obstacle in achieving confirmed diagnoses or reliable serosurveys in endemic areas where other flaviviruses circulate (e.g. dengue, Zika, St. Louis encephalitis, or West Nile viruses). Seroneutralization is considered as the most specific serological technique. However, the assays are laborious and time consuming and are only available in expert laboratories. But the availability of commercial assays for the serological diagnosis of YF has increased the implementation of such techniques. Commercial assays are, in general, thoroughly standardized and offer good standards of sensitivity, specificity, and reproducibility in diagnostic laboratories in order to receive the authorisation for in vitro diagnostics.

The performance of the different techniques applied in YF diagnosis may vary between laboratories, and so far no external quality assessment (EQA) studies addressing their accomplishment have been performed. This international EQA is an important tool to evaluate the performance of protocols currently in use in diagnostic laboratories and to highlight weaknesses in their methodologies and operating procedures.

## Materials and Methods

### Participants

Institutions involved in laboratory diagnostics of YFV infection were invited to participate in this study. Invitees consisted of members of the European Network of “Imported” Viral Diseases (ENIVD), national/regional YFV reference laboratories, and diagnostic laboratories contacted through the Pan American Health Organization (PAHO) or the African Network of Laboratories for polioviruses and hemorrhagic fevers diagnosis. The study was announced as an EQA study on YFV molecular and serological diagnostic methods proficiency, which included certifying and publishing the results in a comparative and anonymous manner.

Thirty-six laboratories from Europe (n = 28), the Americas (n = 7), Middle East Asia (n = 1), and Africa (n = 1) participated in these EQA activities, and reports including 32 and 31 data sets of results were returned for the molecular (28 laboratories) and serological (28 laboratories) diagnosis EQAs, respectively.

Twenty laboratories participated both in the molecular diagnostics EQA and the serological diagnostics EQA from Europe (n = 15), the Americas (n = 4), and Africa (n = 1): IRBA-IMTSSA, Marseille, France; Bernhard-Nocht-Institut, Hamburg, Germany; Institut für Mikrobiologie der Bundeswehr, Munich, Germany; Aristotle University of Thessaloniki, School of Medicine, Greece; Istituto Nazionale per le Malattie Infettive “L. Spallanzani”, Rome, Italy; Fondazione IRCCS Policlinico San Mateo, Pavia, Italy; Norwegian Institute of Public Health, Oslo, Norway; CEVD/INS, Aguas de Moura, Portugal; Institute of Microbiology and Immunology, University of Ljubljana, Ljubljana, Slovenia; Instituto de Salud Carlos III, Madrid, Spain; Hospital Clínic i Provincial de Barcelona, Barcelona, Spain; Spiez Laboratory, Spiez, Switzerland; University of Geneva, Geneva, Switzerland; Erasmus Medical Centre, Rotterdam, The Netherlands; Health Protection Agency, CEPR, Porton Down, Salisbury, United Kingdom; Centro Nacional de Enfermedades Tropicales, CENETROP, Santa Cruz, Bolivia; Instituto Nacional de Salud, Bogotá, Colombia; Institut Pasteur de la Guyane, Cayenne Cedex, French Guiana; Laboratorio Central de Salud Pública, Asunción, Paraguay; Special Pathogens Unit, National Institute for Communicable Diseases, National Health Laboratory Service, SPU/NICD-NHLS, Johannesburg, South Africa.

Eight laboratories participated exclusively in the YFV molecular diagnostics EQA, from Europe (n = 7) and the Middle East (n = 1): Institute of Virology, Medical University Vienna, Vienna, Austria; Slovak Academy of Science, Bratislava, Slovakia; Assistance Publique-Hôpitaux de Marseilles, Hôpital de la Timone, AP-HM TIMONE, Marseille, France; Army Medical and Veterinary Research Center, Rome, Italy; Centre for Biothreat Preparedness, Helsinki, Finland; Universitätsklinikum Freiburg, Freiburg, Germany; Institut für Virologie, Marburg, Germany; National Center for Zoonotic Viruses, MOH-PHL, Tel-Hashomer, Israel.

Eight laboratories participated exclusively in the serological diagnosis EQA, from Europe (n = 5) and the Americas (n = 3); Instituut voor Tropische Geneeskunde, Antwerp, Belgium; Institute of Public Health, Ostrava, Czech Republic; Euroimmun AG, Lübeck, Germany; National Center for Epidemiology, Budapest, Hungary; Crucell Switzerland AG, Berne, Switzerland; Bio Manguinhos, Rio de Janeiro, Brazil; Viral Zoonoses National Microbiology Laboratory, Winnipeg, Manitoba, Canada; Instituto Nacional de Higiene y Medicina Tropical “LIP”, Guayaquil, Ecuador.

The European Network for the Diagnostics of ‘Imported’ Viral Diseases -Collaborative Laboratory Response Network (ENIVD-CLRN) established and coordinated this EQA as in other EQAs previously performed [9]–[12].

### Specimen preparation

The molecular diagnosis EQA panel consisted of inactivated YFV preparations generated from Vero E6 cell culture supernatants infected with different YFV strains: the vaccine strain (YFV-17D), a South American strain (Brazil), and a West African strain (IvoryC1999) [13]. Supernatants were inactivated by heating for 1 h at 56°C and by gamma irradiation (25 kilogray [kGy]) to ensure their non-infectivity. The inactivated supernatant viral load was estimated after heat inactivation and additionally after gamma irradiation by an in-house real-time RT-qPCR with a 95% detection limit in copy number estimated in 6.48 copies/reaction (rxn) (95% CI: 2.35–235 copies/rxn) (C. Domingo, unpublished results).

The inactivated material was diluted in serum plasma to prepare a set of ten positive samples that included five serial 10-fold dilution series of YFV-17D (3×10^6^ Genome equivalents [GE]/sample to 3×10^2^ GE/sample), two YFV (Brazil) dilutions (10^4^ GE/sample and 10^3^ GE/sample), and three YFV (strain IvoryC1999; GenBank Acc. No.: AY603338) dilutions (2×10^4^ GE/sample to 69 GE/sample). As specificity controls we prepared two additional plasma samples, one of them containing West Nile virus (WNV [New York]), Japanese encephalitis virus (JEV [strain SA-14-02]), St. Louis encephalitis virus (SLEV [Parton]), and tick-borne encephalitis virus (TBEV [strain Absettarov]). The second plasma sample contained the four dengue serotypes (DENV-1 VR344 [strain Thai 1958], DENV-2 VR345 [TH-36 strain], DENV-3 VR216 [H87 strain], and DENV-4 VR217 [H241 strain]). Two negative control plasma samples were also included (Table 1).

**Table 1:** EQA results of the 28 participant laboratories in the molecular diagnosis panel.

Sample ID		#2	#9	#12	#4	#14	#10	#5	#13	#1	#6	#11	#3	#8	#7			
Viral load in sample		3×10^6^	3×10^5^	3×10^4^	3×10^3^	3×10^2^	10^4^	10^3^	2×10^4^	2×10^3^	69	NEG	NEG	NEG	NEG			
Lab. no.	RT-PCR technique	17D	17D	17D	17D	17D	Brazil	Brazil	Ivory Coast	Ivory Coast	Ivory Coast	SLEV/JEVWNV/TBEV	DENV1-4	neg	neg	TARGET	Score*	Classification
16a	TaqMan RT-PCR^a^	+	+	+	+	+	+	+	+	+	*(−)*	−	−	−	−	YFV	26	OPTIMAL
8	TaqMan RT-PCR^b^	+	+	+	+	*(−)*	+	+	+	+	*(−)*	−	−	−	−	PANFLAV	24¶	OPTIMAL
17b	RT nested PCR^c^	+	+	+	+	+	+	+	+	+	+	−	*(+)*	−	−	PANFLAV	24¶	NON OPTIMAL
1	TaqMan RT-PCR^a^	+	+	+	+	+	+	+	+	+	+	−	*(+)*	−	−	YFV	24	NON OPTIMAL
27	TaqMan RT-PCR^d^	+	+	+	+	+	+	+	+	+	+	−	*(+)*	−	−	YFV	24	NON OPTIMAL
28	TaqMan RT-PCR^e^	+	+	+	+	+	+	+	+	+	+	−	*(+)*	−	−	YFV	24	NON OPTIMAL
15	TaqMan RT-PCR^a^	+	+	+	+	+	+	+	+	+	*(−)*	−	*(+)*	−	−	YFV	22	NON OPTIMAL
17a	TaqMan RT-PCR^f^	+	+	+	+	+	+	+	+	+	*(−)*	−	*(+)*	−	−	YFV	22	NON OPTIMAL
6	RT nested PCR^x^	+	+	*(−)*	*(−)*	*(−)*	+	+	+	+	*(−)*	−	−	−	−	PANFLAV	20¶	OPTIMAL
22a	RT nested PCR^g^	+	+	ND	+	*(−)*	+	+	*(−)*	*(−)*	+	−	−	−	−	PANFLAV	20¶	OPTIMAL
16b	Heminest Rt-PCR^c^	+	+	+	+	+	+	+	+	+	*(−)*	−	*(+)*	−	−	PANFLAV	20¶	NON OPTIMAL
2	TaqMan RT-PCR^a^	+	+	+	+	*(−)*	+	+	+	+	*(−)*	−	*(+)*	−	−	YFV	20	NON OPTIMAL
9	TaqMan RT-PCR^a^	+	+	+	+	*(−)*	+	+	+	+	*(−)*	−	*(+)*	−	−	YFV	20	NON OPTIMAL
14	RT nested PCR^g^	+	+	+	*(−)*	*(−)*	+	*(−)*	+	*(−)*	*(−)*	−	−	−	−	PANFLAV	18¶	OPTIMAL
4	TaqMan RT-PCR^a^	+	+	+	+	*(−)*	+	+	+	*(−)*	*(−)*	−	*(+)*	−	−	YFV	18	NON OPTIMAL
10	TaqMan RT-PCR^e^	+	+	+	+	+	*(−)*	*(−)*	*(−)*	*(−)*	*(−)*	−	*(+)*	−	−	YFV	14	NON OPTIMAL
11	RT-PCR^h^	+	+	+	+	+	*(−)*	*(−)*	*(−)*	*(−)*	*(−)*	−	*(+)*	−	−	YFV	14	NON OPTIMAL
20	Heminest RT-PCR^c^	+	+	+	+	+	+	+	+	+	+	*(+)*	*(+)*	−	*(+)*	PANFLAV	16¶	NON OPTIMAL
3b	TaqMan RT-PCR^e^	+	+	+	+	+	*(−)*	*(−)*	*(−)*	*(−)*	*(−)*	−	*(+)*	−	−	YFV	14	NON OPTIMAL
5	TaqMan RT-PCR^x^	+	+	+	+	+	*(−)*	*(−)*	*(−)*	*(−)*	*(−)*	−	*(+)*	−	−	YFV	14	NON OPTIMAL
13	TaqMan RT-PCR^a^	+	+	ND	+	+	*(−)*	*(−)*	+	*(−)*	*(−)*	−	*(+)*	−	−	YFV	14	NON OPTIMAL
18	TaqMan RT-PCR^e^	+	+	+	+	+	*(−)*	*(−)*	*(−)*	*(−)*	*(−)*	*(+)*	−	−	−	YFV	14	NON OPTIMAL
3a	RT nested PCR^f^	+	+	+	*(−)*	*(−)*	+	*(−)*	*(−)*	*(−)*	*(−)*	−	*(+)*	−	−	YFV	12	NON OPTIMAL
19	TaqMan RT-PCR^a^	+	+	+	*(−)*	*(−)*	+	*(−)*	*(−)*	*(−)*	*(−)*	−	*(+)*	−	−	YFV	12	NON OPTIMAL
22b	TaqMan RT-PCR^e^	+	+	ND	+	+	*(−)*	*(−)*	*(−)*	*(−)*	*(−)*	−	*(+)*	−	−	YFV	12	NON OPTIMAL
7	TaqMan RT-PCR^i^	+	+	+	+	+	+	*(−)*	*(−)*	*(−)*	+	*(+)*	*(+)*	−	*(+)*	YFV	10	NON OPTIMAL
21	TaqMan RT-PCR^e^	+	+	+	*(−)*	*(−)*	*(−)*	*(−)*	*(−)*	*(−)*	*(−)*	−	*(+)*	−	−	YFV	10	NON OPTIMAL
25	RT-nested PCR^x^	+	*(−)*	*(−)*	*(−)*	*(−)*	ND	*(−)*	*(−)*	ND	*(−)*	−	−	−	−	YFV	8	NON OPTIMAL
12	SYBR-RT-PCR^b^	+	+	+	*(−)*	*(−)*	+	*(−)*	*(−)*	+	*(−)*	*(+)*	*(+)*	*(+)*	−	PANFLAV	6	NON OPTIMAL
23	RT-PCR^j^	+	+	+	*(−)*	*(−)*	+	*(−)*	*(−)*	*(−)*	*(−)*	−	*(+)*	−	−	PANFLAV	0	NON OPTIMAL
24	RT-nested PCR^k^	*(−)*	*(−)*	*(−)*	*(−)*	*(−)*	*(−)*	*(−)*	*(−)*	*(−)*	*(−)*	−	−	−	−	YFV	NS	NON OPTIMAL
26	RT-nested PCR^f^	+	*(−)*	*(−)*	+	*(−)*	*(−)*	+	*(−)*	+	+	*(+)*	*(+)*	*(+)*	*(+)*	YFV	NS	NON OPTIMAL
CORRECT RESULTS (%)		**96.8**	**90.6**	**86.2**	**71.8**	**53.1**	**67.7**	**50**	**50**	**48.3**	**25**	**84.3**	**25**	**93.7**	**93.7**			

* The score reflects the quality of the diagnostic results: +, positive result (score 2 points); *(+)*, false positive (score -2 points); −, correct negative result (score 2 points); *(−)*, false negative (score 0 points).

a [20];

b adapted from [23];

c [19];

d [24];

e [21];

f in-house;

g [18];

h [22];

i [16];

j [15];

k [17];

x not provided.

¶ Sequencing performed; NS: Negative Score; ND: not done; PANFLAV: panflavivirus; YFV: yellow fever virus.

Sample preparations were tested by an in-house real-time quantitative RT-PCR to validate the quality of the samples.

For the serological diagnosis, a panel of 13 samples was prepared by diluting anti-YFV-positive sera from YF vaccinees and from wild-type YFV infections with fresh frozen plasma previously confirmed as negative for flaviviruses. After dilution, the samples were heat inactivated (56°C, 1 h). The proficiency panel consisted of a set of nine samples which included four serial 2-fold dilutions of YFV-17D-positive sera (IgM and IgG positive), two dilutions from a West African wild-type YFV infection serum (IgM negative, IgG positive), and three dilutions from a positive South American wild-type YFV infection serum (IgM and IgG positive). As specificity controls, we included aliquots of two sera containing IgM and IgG antibodies reactive for other flaviviruses (WNV, DENV) and two additional negative sera as controls (Table 2).

**Table 2:** EQA results of the 28 participating laboratories in the serological panel.

Sample ID		#6	#11	#7	4#	#5	#10	#14	#13	#1	#2	#9z	#8	#12		
		αYF17D	αYF17D	αYF17D	αYF17D	αYF-AFR	αYF-AFR	αYF-SA	αYF-SA	αYF-SA	αWNV	αDEN	neg	neg		
Dilution		1∶5	1∶10	1∶20	1∶40	1∶10	1∶20	1∶20	1∶40	1∶80	1∶20	1∶20			Score*	
Lab. no.	Assay	IgM/IgG+/+	IgM/IgG+/+	IgM/IgG+/+	IgM/IgG+/−	IgM/IgG−/+	IgM/IgG−/−	IgM/IgG+/+	IgM/IgG+/+	IgM/IgG+/+	IgM/IgG−/−	IgM/IgG−/−	IgM/IgG−/−	IgM/IgG−/−	IgG	IgM
18	IIF^a^	−/+	−/+	−/−	−/−	−/−	−/−	+/+	+/+	−/+	−/−	−/−	−/−	−/−	22	16
12b	IIF^a^	−/+	−/+	−/−	−/−	−/−	−/−	+/+	+/+	+/+	−/−	−/−	−/−	−/−	22	18
27	IIF	+/+	−/+	−/−	−/−	−/−	−/−	+/+	+/+	+/+	−/−	−/−	−/−	−/−	22	20
4	IIF^a^	−/+	−/+	−/+	−/−	−/−	−/−	+/+	+/+	+/+	−/−	−/+	−/−	−/−	20	18
6	IIF^a^	−/+	−/−	−/eq	−/−	−/−	−/−	+/+	+/+	eq/+	−/eq	−/−	−/−	−/−	20	18
7	IIF^c^	−/+	−/+	−/−	−/−	−/−	−/−	+/+	+/+	eq/+	−/−	−/−	−/−	−/+	20	18
8	IIF^a^	+/+	−/+	−/−	−/−	−/−	−/−	+/+	−/+	+/−	−/−	−/−	−/−	−/−	20	18
29	IIF^a^	+/+	−/+	−/−	−/−	−/−	−/−	+/+	+/+	+/−	−/−	−/−	−/−	−/−	20	20
10	IIF^b^	+/+	+/+	+/+	−/+	−/−	−/−	+/+	+/−	−/−	−/−	−/−	−/−	−/−	20	22
5	IIF^b^	+/+	+/−	−/−	−/−	−/−	−/−	+/+	+/+	+/+	−/−	−/−	+/−	+/+	18	18
20a	IIF	+/+	−/−	−/−	−/−	−/−	−/−	+/+	+/+	−/−	−/−	−/−	−/−	−/−	18	18
3	IIF^a^	+/−	+/−	+/−	−/−	−/−	−/−	+/+	+/+	+/+	−/−	−/−	+/−	−/−	18	22
2	IIF^b^	nd/+	nd/+	nd/−	nd/−	nd/−	nd/−	nd/+	nd/+	nd/+	nd/+	nd/+	nd/−	nd/−	18	nd
25	IIF^a^	+/+	eq/−	−/−	−/eq	−/−	−/−	+/+	+/+	eq/−	eq/−	−/−	eq/−	−/−	16	18
12a	IIF^b^	nd/−	nd/−	nd/−	nd/−	nd/−	nd/−	nd/+	nd/+	nd/−	nd/−	nd/−	nd/−	nd/−	16	nd
13	IIF^d^	nd/−	nd/−	nd/−	nd/−	nd/−	nd/−	nd/+	nd/+	nd/−	nd/−	nd/−	nd/−	nd/−	16	nd
28	IIF^a^	−/−	−/−	−/−	−/−	−/−	−/−	+/+	+/−	−/−	−/−	−/−	−/−	−/−	14	16
14a	IIF^a^	+/−	−/−	−/−	−/−	−/−	−/−	+/+	+/−	+/−	−/−	−/−	−/−	−/−	14	20
11	IIF^b^	nd/−	nd/−	nd/−	nd/−	nd/−	nd/−	nd/+	nd/−	nd/−	nd/−	nd/−	nd/−	nd/−	14	nd
1	IIF^a^	−/−	−/−	−/−	−/−	−/−	−/−	+/−	+/−	−/−	−/−	−/−	−/−	−/−	12	16
16	ELISA^f^^,^ g	−/−	−/−	−/−	−/−	−/−	−/−	+/−	+/−	+/−	−/−	−/−	−/−	−/−	12	18
19	ELISA^b^	+/eq	+/−	+/+	eq/+	−/−	−/−	+/−	+/−	−/+	+/+	−/+	+/+	−/+	10	20
26	ELISA^b^	−/nd	−/nd	−/nd	nd/nd	−/nd	−/nd	nd/nd	+/nd	−/nd	−/nd	−/nd	−/nd	−/nd	nd	14
23	ELISA	−/nd	−/nd	−/nd	−/nd	−/nd	−/nd	+/nd	+/nd	+/nd	−/nd	−/nd	−/nd	−/nd	nd	18
22	ELISA^e^	eq/nd	eq/nd	−/nd	−/nd	−/nd	−/nd	+/nd	+/nd	+/nd	−/nd	−/nd	−/nd	−/nd	nd	22
24	NT	+	+	+	+	−	−	+	+	+	−	−	−	−	24	
30	NT	+	+	+	+	+	−	+	+	+	−	−	−	+	24	
9	NT	+	+	+	−	−	−	+	−	−	−	−	−	−	18	
15	NT	+	+	+	−	+	−	−	+	+	−	−	+	+	18	
14b	NT	+	+	−	−	−	−	+	−	−	−	−	−	−	16	
20b	HAI	<1∶20	<1∶20	<1∶20	<1∶20	<1∶20	<1∶20	1∶20	<1∶20	<1∶20	<1∶20	<1∶20	<1∶20	<1∶20	6	

The score reflects the quality of the diagnostic results: +, positive results; −, negative result; nd, not done; eq, equivocal. NT, seroneutralization test; HAI, haemagglutination inhibition test; Wrong results are marked in grey.

a EUROIMMUN;

b In-house assay;

c [25];

d Bernhard Nocht Institut für Tropenmedizin, Hamburg;

e [32];

f [33];

g [34].

For both panels, aliquots of 100 µl each were number-coded, freeze dried for 24 h (Christ, AlphaI-5, Hanau, Germany) and stored at 4°C until dispatch.

### Validation of the panel sets and dispatch

To validate the molecular panel, we tested three different sets of EQA samples before distribution by the Robert Koch Institute (RKI), Berlin, Germany. After reconstitution with 100 µl of water, the samples were extracted using the QIAamp viral RNA minikit (Qiagen, Hilden, Germany) according to the manufacturer's instructions. As mentioned above, we estimated the YFV genome copies present in these samples by an in-house real-time RT-PCR (Table 1).

Similarly, two sets of samples for the serological EQA panel were validated before distribution. After reconstitution with 100 µl of water, samples were analyzed by IIF for the presence of specific YFV antibodies using commercially available kits (FK 2665-1010-G and FK 2665-1010-M, Anti-yellow fever virus IIFT, Euroimmun, Lübeck, Germany) following the manufacturer's instructions. These assays have demonstrated a specificity of 94.7% and 96.7% in the detection of IgG and IgM antibodies in two panels of patient sera involving 300 and 294 sera each. Similarly, their sensitivity was determined as 94.7% for IgG and 94.4% for IgM (stated by manufacturer). A microneutralization assay in Vero E6 cells was also carried out to confirm the presence and specificity of the antibodies as previously described [14].

The EQA panels were distributed to participants with full instructions. Samples were shipped at ambient temperature by post to participating laboratories. We requested participant laboratories to resuspend the samples in 100 µl of water and to analyse the material as serum samples for YFV molecular/serological diagnosis as done routinely. They were asked to report their results and any problems encountered, as well as to provide information on the protocol details (RT-PCR method, extraction procedure, serological methods, sera dilution) using a common form included in the documentation.

### Evaluation of the results

To assure anonymous participation, an individual numerical identification code was assigned to the results sent by each laboratory. This number was followed by a letter (a, b) in case different laboratory results were received based on different methods.

A scoring system was established for sensitivity and specificity obtained by each participant laboratory. For the molecular diagnostics EQA evaluation we assigned two points for correct results (100% = 28), and penalised false-positive results with -2 points. We considered those methods as non-optimal which failed to detect one or more strains of YFV, or presented false-positive results in the negative samples. In those cases when a false-positive amplification result was obtained by RT-PCR in the “non-specificity control” samples (#3 and #11), which were however correctly identified by sequencing, the result was considered correct.

For the serological diagnostics EQA evaluation we also assigned two points for correct results (100% = 26), whereas false-negative/-positive results were not scored. Equivocal or borderline results were considered as positive. IgM and IgG results were considered separately.

The complete panel of results was sent to the participants in an anonymous manner where they could only identify the results from their own laboratory.

### Statistical Analysis

Data collected were entered into Microsoft Excel (Microsoft Corp., Bellingham, WA, USA) and analysed using SPSS 14.0 for Windows (SPSS Inc., Chicago, IL, USA).

Logit analysis was used to evaluate the effect of viral RNA concentration on the RT-PCR performance by using cumulative fractions of positive results reported for each test sample of the 10-fold dilution series of YFV-17D. The result reflects the performance of a hypothetical average laboratory.

Results with respect to categorised variables were analysed by McNemar's test. T-test and Mann-Whitney tests were used to estimate the effect of the real-time RT-PCR format on the performance. P-value<0.05 was considered to indicate statistical significance.

## Results

A total of 36 laboratories participated in this EQA. Among the participants, 20 laboratories (56%) reported both serological and molecular results, indicating that they included both approaches in their diagnostic algorithm. However, 22% (8 out of 36) of the participants only applied either molecular or serological techniques, respectively.

A total of 32 laboratory results were received for the molecular diagnosis study (Table 1), including four double sets from laboratories using two methods each (sets 3ab, 16ab, 17ab, 22ab). A total of 31 laboratory results were received for the serological diagnosis study (Table 2), including three double sets from laboratories using two methods each (sets 12ab, 14ab, 20ab).

### Molecular diagnosis results

A variety of tests were used for screening and identification of YFV genome by participating laboratories; these included RT-PCR (n = 2, 6% of the laboratory results) [15], [16], RT-nested PCR (n = 8, 25%) [15], [17], [18], hemi-nested RT-PCR (n = 2, 6%) [19], TaqMan (n = 19, 60%) [16], [20]–[24], and SYBR Green [23] (n = 1, 3%)-based real-time RT-PCR assays (Table 1). As many as ten published protocols (indicated in Table 1) were used by participants and only three methods were established “in house” (9.37%). Eight laboratories used the TaqMan RT-PCR developed by Drosten et al. [20], and six of them reported using the TaqMan RT-PCR developed by Bae et al. [21], in both cases with varying performance depending on the reporting laboratory.

Performance varied among the 28 laboratories (Table 1). Five out of 32 (16%) analyses reported met all criteria with optimal performance; 13 out of 32 (41%) test results achieved non-optimal results due to the failure to detect one or more YFV strains (in 12 sets of results wild-type strains were not detected, whereas in one data set the YF-17D strain was not detected). One laboratory did not report any positive results. Additionally, 78% (25 out of 32) of laboratory results reported false positives; 21 laboratory reports falsely identified YFV in samples containing other flaviviruses (#3 and #11) and four do so in negative samples (Table 1).

**Figure 1:**
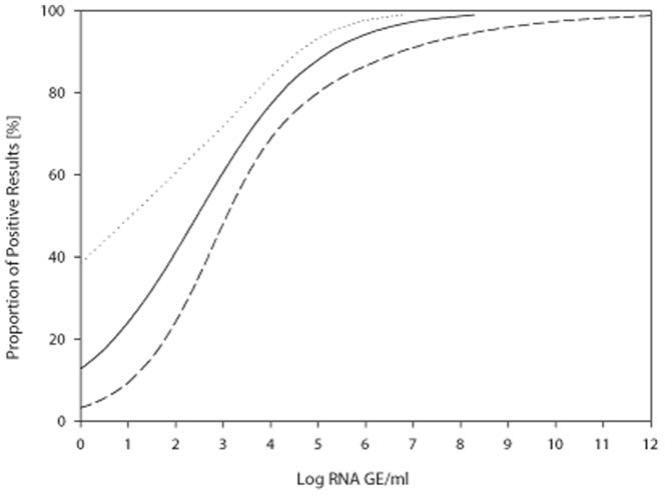
Logit analysis of laboratory tests with a correct result (y axis) for YFV-17D related to viral RNA concentration in positive samples (x axis). Data points represent individual samples in the panel. Thick line is the regression line calculated on the basis of a logit model (dose–response curve), and thin lines are 95% confidence intervals.

Serial dilutions of YFV-17D (samples #2, #9, #12, #4, and #14) were used in order to test the sensitivity of the different methods. To estimate the effect of virus concentration on RT-PCR performance, Logit analysis was carried out using cumulative RT-PCR-positive results reported for each sample of the 10-fold YFV-17D dilution series (Figure 1). The data demonstrated that 50% of positive performance could be expected at a concentration of 2.8×10^2^ GE/sample (95% confidence interval [CI] 11.7–1.2×10^3^ GE/sample). Ninety-five percent performance could be expected for 1.5×10^6^ GE/sample (95% CI 1.9×10^5^–2.8×10^8^ GE/sample).

Comparison of the results obtained for similar concentrations of different YFV strains (Table 1) also suggests the effect of virus type on the performance. Thus the results for YFV-17D were significantly better than those for the IvoryC1999 strain (McNemar's test was performed for sample #12 versus sample #13 and for sample #4 versus sample #1). The performance for YFV-17D was also significantly higher than that for the Brazilian strain (McNemar's test performed for sample #12 versus sample #10 and for sample #4 versus the sample #5). At the same time no essential difference in test performance was found when comparing Brazilian to Ivory Coast YFV samples.

Only four laboratories used two different molecular techniques for the evaluation, an advantageous approach to exclude false positives/negatives in routine diagnosis. The four laboratories used a combination of TaqMan RT-PCR and RT-nested PCR techniques. Laboratories no. 3 and 16 achieved better scores when using the TaqMan protocols, whereas laboratories no. 17 and 22 did with the RT-nested PCR ones (Table 1).

Logit analysis was also carried out separately for results obtained with different RT-PCR techniques. For TaqMan-based real-time RT-PCR methods 50% performance could be expected when viral genome concentration was equal to 1.6×10^2^ GE/sample (95% CI [0.1–584]) while for the RT nested PCR protocols it was achieved at 5×10^3^ GE/sample (95% CI [87–3.7×10^4^]). 95% of certainty was achieved with TaqMan real-time RT-PCR at 1.1×10^4^ GE/sample (95% CI [3×10^3^–2×10^7^]), compared to RT nested PCR at 1×10^8^ GE/sample (95% CI [2.4×10^6^–2.8×10^15^]), suggesting a better sensitivity of the TaqMan protocols. However, t-test comparing the TaqMan real-time methods' performance to that of RT nested PCR protocols did not reveal any significant difference in performance of the methods applied. In general, it seemed that the success of the analysis depended rather on the performance of the individual laboratories than of the format of the RT-PCR.

We requested further information on the number of copies of YFV genome in the samples sent to the participants in order to estimate the laboratories' experience in viral load determination. Only eight results out of 32 (25%) reported quantitative results (data not shown), although 20 laboratories reported results obtained by real-time-based procedures which are suitable to provide quantitative data. Among the reported results, five provided the quantification estimation as Ct values which give very limited data to accurately estimate the samples' viral load in the absence of calibrated standards.

Another point to consider in view of the results reported is the use of methods for the generic detection of flavivirus genome. Nine laboratories reported results using different pan-flavivirus protocols [18]–[20], [23], and five of them correctly differentiated the YFV samples from other flaviviruses in samples #3 and #11 by sequencing. This approach (detection plus sequencing) is suitable for diagnosis purposes as long as a good sensitivity is reached.

Some non-optimal results were due to the presence of false positives, mainly in sample #3 (only 25% of correct results) containing the four dengue serotypes and sample #11 containing other flaviviruses (Table 1). Four laboratories reported false positive results in samples containing only human plasma, which indicates the need to improve laboratory procedures since carry-over contamination must be suspected.

### Serological diagnostics results

Most of the serology laboratory results were obtained by using IIF for the detection of specific YFV antibodies (20 out of 31, 65%), but also ELISA (5 out of 31, 16%), seroneutralization (5 out of 31, 16%), and haemagglutination (1 set of results, 3%) assays.

Performance varied, depending mainly on the method used and the subclass of antibodies to be detected but also on the performing laboratory (Table 2).

Four out of 25 reports (16%) using IIF or ELISA tests did not include results for IgM antibodies, whereas in the case of IgG detection only three laboratories (12%) did not report results, testing only the presence of IgM as marker of infection.

Of the 21 test results for IgM analysis, ten (48%) did not report the presence of anti-YFV-17D IgM antibodies even in the first serial dilution of YFV-17D-positive sera. Among them, six results out of ten (60%) were obtained using a commercial IIF test (EUROIMMUN), three out of ten (30%) using “in-house” ELISA, and one out of ten (10%) using a published IIF protocol [25]. In general, however, in the serial dilutions of samples containing anti-YFV antibodies against the South-American YFV strain, a good sensitivity profile was observed. Only seven out of 21 test results referring to IgM (33%) did not report anti-YF IgM antibodies in sample #1, the sera sample most diluted. Of these, two out seven (28.5%) laboratory results were obtained using an “in-house” ELISA test, two out of seven (28.5%) using an “in-house” IIF method, and three out of seven (42.5%) using a commercial IIF assay (Table 2).

Two laboratories out of 25 (8%) performing IIF or ELISA assays reported false-positive IgM detection in sample #2 which contained antibodies against WNV. However, as many as four laboratories (16%) reported false-positive results in samples #8 and/or #12 (negative control samples).

Regarding anti-YFV IgG results reported during this EQA, a better performance was apparently achieved by laboratories using IIF protocols, as they obtained a higher score in the evaluation (Table 2).

Two laboratories out of the 22 (9%) sending IgG results completely failed to detect IgG antibodies. No laboratory reported the presence of IgG antibodies against YFV (African origin) in samples #5 and #10. In the serial dilution containing anti-YFV antibodies against a South-American YFV strain used as sensitivity control, 86% of the data reported the presence of anti-YFV IgG antibodies in sample #14, 68% in sample #13, and 45.5% in sample #1. However, in eight test results (36%) the presence of anti-YFV IgG (17D) was not detected in the lowest serial dilution of the YFV-17D-positive sera. Among these, four laboratories out of eight (50%) used a commercial IIF assay (EUROIMMUN), two (25%) an “in-house” IIF test, one (12.5%) an IIF assay purchased from the Bernhard-Nocht-Institut (Hamburg), and one (12.5%) used two-in house ELISAs. In 13 reports (59%) IgG detection in YFV-17D samples failed for sample #11, and in 18 reports (82%) for sample #7 (highest dilution). Unexpectedly, none of the laboratories using anti-YFV ELISA detected the presence of IgG properly. Laboratory no. 19 reported the detection of IgG in samples #1 and #7 but not in previous dilutions of the same samples.

False IgG positives were reported in six laboratory results, three of them in samples #2 and #9 containing antibodies against other flaviviruses, and another three in sample #12, a negative control serum. The fact that other laboratories using the same commercial assays did not report these false positives results, suggests a problem in the operational procedures rather than a non-specific cross-reactivity of the assays.

In general, no difference in performance was found for IIF and ELISA assays or for the usage of commercial versus “in-house” assays (data not shown), nor was a difference found in the detection accuracy for IgM and IgG anti-YFV antibodies (McNemar's test).

The data obtained by seroneutralization assays do not allow to distinguish among IgM and IgG antibodies since the assay determines the presence of total neutralizing antibodies. Seroneutralization assays are expected to be the most specific ones in flavivirus serological diagnostics. In this study they showed a good performance with high scores (Table 2). Indeed, even in the low-titre sample #5 (IgM−/IgG+) laboratories nos. 15 and 30 reported a correct positive result. However, the presence of false-positive results for samples #8 and #12 raises the question whether these results are due to a higher sensitivity or a strong background in the test. Control samples for the cytotoxicity effect and controls for virus infectivity must be included in each assay to be able to interpret the results correctly.

The haemagglutination assay which was only used by laboratory no. 20 showed a very low sensitivity, with only sample #14 reported as positive (Table 2).

## Discussion

This is the first international EQA on YF diagnostics. The increasing importance of this disease in Africa and the Americas, and the risk of expansion to other areas, makes it necessary to assure that the methods used for YFV diagnostics and surveillance are working properly where they are already implemented.

Among the participating laboratories 20 out of 36 (56%) reported both serological and molecular results, indicating that they routinely include both approaches in their diagnostic algorithm. However, 22% (eight out of 36) of the participants applied only molecular or serological techniques, respectively, leaving room for the presence of false-negative results depending not only on the accuracy of the methods but also on the time period between the onset of disease and the diagnosis. We recommend combining both molecular and serological methods to provide the best accuracy in the diagnosis of wild-type YFV infections and YFVAE, an approach which increases the diagnosis window while minimizing the risk of false-negatives results. Likewise we also recommend the use of paired samples, when possible, to validate the results obtained in acute samples by confirming the presence/absence of seroconversion. This strategy is useful for the detection of false positives due to cross-contaminations in the case of molecular diagnosis or false isolated IgM positives in the case of serological diagnosis.

Regarding molecular diagnosis EQA, the participants using TaqMan real-time RT-PCR-based techniques overall showed a better performance with a higher sensitivity than other assays. However, the major limitation for the implementation of these assays is the costs of both thermocyclers and reagents which hamper a generalized application in the field.

One of the main weaknesses observed during this EQA was the inability of some protocols to detect the YFV genome of wild-type strains. Five out of eight laboratories which failed to recognize all wild-type YFV strains used the TaqMan real-time RT-PCR described by Bae et al. [21], two out of eight used “in-house” methods, and one applied the method previously described by Brown et al. [22] who already pointed out that false negatives might occur when using this protocol. One laboratory used a TaqMan real-time RT-PCR previously described exclusively for the detection of YFV vaccine strain 17D [16], which would explain the results obtained. However, the methods mentioned above showed a very good sensitivity profile against YFV-17D, making them useful tools to identify viral genome in suspected YFVAE or research, but obviously not to identify suspected wild-type cases. The presence of mismatches between the oligonucleotide and the viral target sequences might explain the failure in genome amplification. This denotes the need to adapt and up-date regularly oligonucleotide sequences in use in diagnosis laboratories to detect the presence of mutations in the circulating strains which may compromise the ability of the assay to amplify and/or detect the targeted sequence leading to false negative results. However, among those laboratories which failed to detect only one of the wild-type strains (South American [Brazil] or West African [IvoryC1999]), two laboratories reported the use of the TaqMan real-time RT-PCR described by Drosten et al. [20], one laboratory used the RT-nested PCR by Kuno [15], and one laboratory used an “in-house” protocol. Other participants applied the method published by Drosten et al., with optimal detection of all YFV strains. Therefore, it seems in these cases that failure in detecting some strains might more likely be due to the specific performance of the laboratories rather than to the techniques themselves, suggesting the need to revise the running protocols of these laboratories to improve the quality of their results.

One laboratory completely failed in the detection of all positive samples. It can not be excluded that the gamma irradiation of the samples for inactivation could have resulted in nicked RNA that would affect those methods amplifying fragments of around 600 bp [26] as it is the case of the method used by this laboratory [17]. However, other laboratories reported good results with bigger amplification targets so probably other factors might have influenced the performance of this laboratory.

Similarly, the presence of false-positive results in flavivirus RNA-free samples also denoted the need to optimize laboratory practices in order to avoid the occurrence of cross-contamination.

It is remarkable that for the sample containing genome of the four dengue serotypes (sample #3) only 25% of the results were correct. In the meantime the possibility of the presence of contaminant traces of YFV genome due to the processing and preparation of the samples has been excluded, and these results could indicate some degree of non-specificity in the techniques. As dengue is a common differential diagnosis for YFV, such specificity issues should be taken into consideration while interpreting dengue or YFV diagnostic testing results.

Twenty-eight percent of the tests reported used a generic flavivirus assay for the detection of YFV RNA, and 55.5% of these correctly differentiated the presence of YFV genome from that of other flaviviruses by consecutive sequencing. This approach might be advantageous for diagnostic purposes in laboratories which need to cover the possibility of infections with different flaviviruses without holding available a specific protocol for each of them. However, we must point out that the use of these generic methods must always be accompanied by sequence identification, mainly in endemic areas where usually more than one of the flaviviruses circulates.

As in previous EQA studies, data regarding viral load were scarce even though the use of real-time techniques was prominent [12].

Regarding the serological results of this EQA, one of the main observations was the fact that 16% of the participating laboratories do not routinely include the analysis of specific anti-YFV IgM in their diagnostic algorithms. It is well known that for the serological diagnosis of an acute YFV infection it is preferable to test for the presence of IgM which appears for a short period of time soon after the infection, confirming a recent contact with the virus. The presence of IgG does not provide a good proof of recent YFV infection and requires the analysis of a second sample to confirm a rise in the antibody titres. In general, the sole detection of IgG would only confirm a previous contact with YFV, even by vaccination, or with another flavivirus as serological cross-reactivity is more pronounced for IgG detection than for IgM, as suggested by this EQA and previous reports [27]–[29]. It is important to note that in general the existence of this cross-reactivity is the major weakness of the serological diagnosis, not only for YFV but for all flaviviruses. Seroneutralization assays are considered the “gold standard” for specific identification of a positive immune response. However, some degree of non-specificity can not be excluded and such a technique requires the use of well-characterized controls. Additionally, seroneutralization assays can perform poorly in samples with multiple/subsequent infections unless multiple samples are available. Also the material, the expertise, and the time required to perform a seroneutralization assay do not make it a proper choice for early diagnosis.

Fortunately, from the results of this EQA it has become obvious that false positives due to cross-reactivity are not the main limitation of the serological techniques used by the participating laboratories, and that most of the specificity difficulties could be solved by proper standardization of the protocols and the use of adequate controls during the assays.

One of the main issues that deserve more attention is the apparent lack of sensitivity regarding anti-YFV-17D IgM detection in 48% of the results reported, while a good sensitivity profile was observed in wild-type infection sera. This should be taken into account when assessing the protection provided by the YFV vaccines by determining IgM levels.

In the case of IgG detection, apparently the quality of the results depends mostly on the performing laboratories since their results differ even when using the same technique. The lack of detection of IgG in sample #5 has no major impact on the conclusions of this EQA since the samples from West African wild type infection contained the lower titre of antibodies compared to the others, and required very sensitive techniques for detection, which could only be achieved by seroneutralization assays in this EQA. Similarly, the presence of false-positive results in those laboratories with other correct results makes it unclear whether a low threshold of detection could imply a higher risk for false-positive results to occur.

We conclude that the main differences in the molecular diagnosis results might be more related to the handling of assays and specimens, pointing out the importance of regularly revising and improving the operational protocols. The different profiles of strain detection obtained with each protocol must be taken into account by the laboratories which must select those protocols most suitable for their particular diagnosis purpose (wild-type infections vs. YFVAE).

In general, commercial serological assays showed a very good sensitivity profile in this study for both IgM and IgG detection of wild-type origin, but not for antibody detection against the vaccine strain YFV-17D. Commercial serological assays used for diagnostic purposes show a better specificity when compared to “in-house” ones. However, their use raises some concern when evaluating the immune response elicited after vaccination.

No difference in the sensitivity detection of IgM and IgG anti-YF antibodies has been found in this EQA, in contrast with the results observed in previous EQA studies on other arboviral infections [11], [30], [31]. However, the different titres of IgM and IgG in the samples included in this EQA make this comparison quite general.

The low participation of endemic countries in this EQA, even though widely announced, points out the need to encourage more laboratories to implement YFV diagnosis techniques and to participate routinely in quality assurance programmes.
